# Is Senna Laxative Use Associated to Cathartic Colon, Genotoxicity, or Carcinogenicity?

**DOI:** 10.1155/2009/287247

**Published:** 2009-09-10

**Authors:** M. A. Morales, D. Hernández, S. Bustamante, I. Bachiller, A. Rojas

**Affiliations:** ^1^Departamento de Farmacologia, Instituto de Ciencias Biomédicas, Facultad de Medicina, Universidad de Chile, 8380453 Independencia, Santiago, Chile; ^2^Departamento de Ciencias Biológicas, Facultad Ciencias de la Salud, Universidad Andrés Bello, República 217, Santiago, Chile; ^3^Sociedad Asturiana de Fitoterapia, 33005 Oviedo, Asturias, Spain; ^4^Facultad de Ciencias de la Salud, Escuela de Medicina, Universidad Católica del Maule, Casilla 617, Talca, Chile

## Abstract

Due to their natural origin, apparent low oral toxicity, effectiveness, and accessibility without a medical prescription, the anthranoid laxatives are a popular remedy for constipation and are frequently used abusively. Therefore, it is important to characterize its harmful and/or toxic effects. The sennosides, main active metabolites of senna, exhibit a very low toxicity in rats, and its genotoxic activity in bacterial strains as well as mammal cells was classified as weak in those cases where it was shown to be significant. The toxicological and mutagenic status of the crude extract of senna, however, is not as well characterized, and it is necessary to do so since it is frequently, and at the same time incorrectly, believed that the chronic use of anthranoid laxatives is a risk factor for the development of colorectal cancer. The objective of this article was to review the information that arises in various scientific medical databases using key words such as senna, sen, *Senna alexandrina, Cassia angustifolia*, sennosides, laxative toxicity, mainly ISI and non-ISI articles of journals with an editorial committee. Web pages of products or companies that publicize or commercialize this type of laxative were not included. This analysis establishes that (1) there is no convincing evidence that the chronic use of senna has, as a consequence, a structural and/or functional alteration of the enteric nerves or the smooth intestinal muscle, (2) there is no relation between long-term administration of a senna extract and the appearance of gastrointestinal tumors or any other type in rats, (3) senna is not carcinogenic in rats even after a two-year daily dose of up to 300 mg/kg/day, and (4) the current evidence does not show that there is a genotoxic risk for patients who take laxatives containing senna extracts or sennosides.

## 1. Introduction

Our current lifestyle has led to an important increase in the demand for laxatives, medicinal products that induce or contribute to defecation. Their high demand is clearly established: in 1995, it was estimated that 25% of women and 10% of men in Germany considered they had difficulty to defecate and suffered constipation [1]. 

Along with analgesics, laxatives are the most commonly obtained medicines by patients without consulting a doctor. Therefore, they could be easily used inadequately and abusively. In Germany sales figures of around 39 million units per year, with a value of DM 400 million [1], and in the United States around USD 600 million of laxatives are sold every year [2, 3].

The commercialization of laxatives is greater every year and covers broad sectors of modern society, where its use is observed in different economic strata and different age groups. This is a cause for concern and leads to a permanent analysis of risks which could be associated to the massive use of these types of medicines.

Among the most used laxatives being self-administed or prescribed by a physician, we find that senna infusions, bisacodyl, sodium picosulphate, sodium docusate, stimulating laxatives, and lactulose are considered osmotic-type laxatives.

From its introduction by the Arabs in the XIX century, the anthranoid laxatives have been widely used around the world. Among the plants that contain anthranoid laxatives, Senna (*Senna alexandrina* or *Cassia angustifolia*) is the most used. Due to their natural origin, apparent low oral toxicity, effectiveness, and accessibility without a medical prescription, the anthranoid laxatives are a popular medicament for constipation, frequently used abusively. 

There is an important need, therefore, to characterize the potential harmful and/or toxic effects of the anthranoid laxatives. The derivatives are obtained from leaves and/or dried pods of *Senna alexandrina*, or from the direct use of the plant parts, separately or mixed.

The anthranoid compounds are classified in anthrones (rhein anthrone), anthraquinones (aloe-emodin, chrysophanol rhein) and dianthrones (sennosides), and are present in plants mainly as sugar derivatives. Since the *β*-glycosidic bond is resistant to acid digestion and to *α*-glycosidase activity in the small intestine, the anthranoid glycosides are not absorbed in the small intestine. Instead, they are maintained as prodrugs until they reach the large intestine where they are metabolized to the active drug, the aglicone rhein anthrone for the *β*-glycosidase and reductase activity of the intestinal flora [4, 5].

Senna contains various anthranoids, where the most important are sennosides A and B; followed by aloe-emodin, emodin, and chrysophanol [6]. The sennosides, main active metabolite of senna, show a very low toxicity in rats [7] and their genotoxic activity in bacterial strains as well as mammal cells, in the cases where it was significant, was classified as weak [8–11]. 

The toxicological and mutagenic status of the senna crude extract, however, is less characterized. In a study by Hietala et al. [7], the laxative effect and the acute toxicity of certain fractions of senna extracts in rats were investigated. The same tests were carried out with various pure anthraquinone derivatives in senna pods. Results showed that the laxative and toxic components could be separated from pods and senna extracts. The most powerful laxative components, sennosides A + B and the *V* fraction (with relative potentials of 1 and 0.9, resp.) have the lowest toxicity (intravenous toxicity relative to 1 and less than 1) while the fractions with very low laxative activity (rhein-8-glucoside and fraction *IV* with relative potentials of 0.56 and 0.05) have the highest acute toxicity (relative toxicities of 10 and 32, resp.). This suggests that there are other active molecules in the senna extract that could be responsible for its toxicity. Anthranoids such as chrysantine, hidroxyanthraquinones, presenting in trace concentrations in the extract, show a different and highly controversial toxicological status to the sennosides. The hydroxyanthraquinones emodin and aloe-emodin gave positive results in genotoxic assays in *Salmonella typhimurium*, V79-HGPRT, rat hepatocytes, and mouse fibroblasts [12], however, in another study, such genotoxicity was not observed [10]. In a study by Mori, the induction of neoplasms in the intestine, stomach, and rat liver, subjected to a diet containing 1% hidroxyanthraquinones for 480 days, was induced [13]. It is worth mentioning that these effects were only observed at extremely high doses and prolonged treatments. Other studies have not found mutagenic effects for these compounds. However, it is necessary to precisely pinpoint the possible harmful effects of the anthranoid laxatives, since recently and from results by Siegers, it has been suggested that the chronic use of laxatives such as aloe (*Aloe spp*.), frangula (*Rhamnus frangula*), and cascara sagrada (*Rhamnus purshiana* DC) could constitute a risk factor for the development of colorectal cancer [14].

The objective of this article was to review scientific-medical publications found in different databases (Toxnet, Ovid, IBIDS, ScienceDirect, Scirus, PubMed) with keywords such as senna, sen, *Senna alexandrina*, *Cassia angustifolia*, sennosides, and laxative toxicity. Web pages of products and companies that publicize or commercialize these types of laxatives were not included.

## 2. Inhibitory Effects of the Motility and Secretions of the Colon

The oral administration of sennosides (20–30 mg/kg) to fasting dogs induces a strong and prolonged inhibition of the myoelectric activity of the colon that is evident after a delay of 6–10 hours that corresponds to the orocecal transit and to the metabolism in the colon accompanied by an abundant diarrhea. When the sennosides are administered 1 hour prior to feeding, the habitual postprandial increase of colon motility does not appear. The modulation of colon motility by the sennosides has been broadly documented and the appearance of 3 to 10 giant contractions of high amplitude has been confirmed under its effect. These single contractions propagate in the second portion of the colon at a velocity of 0, 5–2 cm/min. The elimination of liquid faecal matter has been associated to these contractions. It has also been confirmed that these contractions are not only produced by sennosides but also by other compounds such as guanethidine, neostigmine, castor oil, and intraluminal hypertonic glucose [15].

There is a direct influence on intestinal epithelial cells after the administration of the sennoside metabolite rhein-anthrone. At low doses, there is a change in cellular form and of the organelles clearly related to an increase in metabolism. High doses of rhein-anthrone induce apoptotic changes. It seems that the interaction between epithelial cells and the monocyte-type cells also induces the release of type E prostaglandins. When rhein-anthrone is added to a group of epithelial cells and mononuclear peripheral blood cells, there is a 140% increase of the control value of PGE2. This finding indicates that rhein-anthrone is activating intestinal immune system components and may induce the secretion and motility [16].

The oral administration of 50 mg/kg of sennosides modifies the net secretion of water, Na^+^ and Cl^−^ after 6 hours, a return to normal values occurs during the next 18 hours. After 24 hours, a significant increase in the secretion of K^+^ and Ca^++^ is also observed. The sennosides do not modify the activity of the Na^+^ − K^+^ pump of the mucosa nor of the phophodiesterase enzyme; the levels of cAMP do not vary either [17].

## 3. Toxicity Background

At a reproductive toxicity level, by 1986, U. Mengs had established that the intragastric administration of sennosides did not induce embryolethal, theratogenic, or fetotoxic effects. In his assays, using rats and rabbits, he also determined that the sennosides did not have an effect on the postnatal development of young animals, on their mother's behavior, nor on the masculine or feminine fertility [18].

In 1987, Hietala et al. [7] investigated the laxative and toxic activity using different fractions of senna on mice. Their results showed that it was possible to identify the laxative components of those components that showed an acute toxic activity. These studies, however, only considered acute toxicity (24 hours) and were carried out using very high doses [19]. The sennosides were classified as not very toxic in rats and mice after administrating an oral dose. The LD50 values were 5 000 mg/kg in both species. Whatever the case, the cause of death was probably due to an intense water and electrolyte loss following an intense diarrhea.

In subacute studies with rats and dogs to which a maximum dose of 20 mg/kg and 500 mg/kg was administered, respectively, the sennosides did not cause local nor systemic toxicity. A low weight increase was observed in the rat kidneys which was irrelevant. In a more prolonged study in rats to which a dose of up to 100 mg/kg was administered, no toxic effects were observed after 6 months [20]. The effects on food consumption, weight gain, and some biochemical parameters, in addition to slight renal lesions, were interpreted as secondary effects that arise as a consequence of a chronic diarrhea. In the same study, no abnormal results were observed in neither mutagenic nor reproductive tests.

Another toxicity study designed a treatment of rats with senna fruit extracts 1, 25, 100, and 300 mg/kg/day for 104 weeks. Based on clinical signs related to the laxative effect, such as the mucosity of the faeces, the 300 mg/kg/day dose was considered to be the maximum tolerated dose [21]. At this dose, the animals reduced their body weight, increased their water consumption, and experienced notable changes of electrolytes with increases in K^+^ and Cl^−^ in the plasma and decreases in Cl^−^, Na^+^, and K^+^ in the urine. The variations in electrolytes are adaptative changes to the laxative effect of senna.

## 4. Morphological Changes

After a gastric administration of 100 mg of sennosides/kg body weight, morphological changes are not observed in the rat colon. In the electronic microscopy examen, no damage in the intramural nervous tissue appeared [22].

According to Dufour and Gendre, in 1988, some animal and human studies revealed damages in the myenteric plexus and the colon epithelium, but they recognized that other studies had revealed the inexistence of such changes under similar treatments [23]. These same authors carried out a histological and ultrastructural study to try and evidence the toxicity of sennosides in rats, acknowledging that they were unable to find intestinal mucosal damage after a long-term treatment. The comparison of the effect of the sennosides and an anthracenic derivative 1,8-dihidroxyanthraquinone on the jejunum and colon of rats showed that only the synthetic compound caused anomalies in the myenteric plexus. This was interpreted as a good tolerance of the intestinal mucosa to sennosides, as opposed to nonglucosidic compounds [23].

Another study investigated the effect of the sennosides on the myenteric plexus of the colon. Rats and mice were treated for 4-5 months with sennosides administered in drinking water. A decrease in the body weight was observed while the colon weight increased in relation to the body weight of the animals tested. The determination of the number of neurons in the rat colon was not affected but an increase in relation to the controls was observed in mice. No evidence was obtained of destruction by toxicity or variation in the neuron population; the appearance of isolated somas or axons was not observed by antibody staining, which would have detached from the myenteric plexus. It was concluded that senna did not destroy the myenteric neurons from the rat or mouse colon [24].

It has been shown that the sennosides induce cytochemical changes in epithelium cells of the cecum, rectum, and colon of rats. After 12 weeks of treatment, an increase in the total acidic content of mucine with a decrease of sulfomucin and increase of sialomucin was observed. An increase in the expression of cytokeratin AE1 was also exhibited. The results were interpreted as being of functional origin, and the association to early precancerous lesions was discarded [25].

A light and electronic microscope study in intestine of Guinea pigs treated for 14 consecutive days with senna, sennosides, danthron, and bisacodyl by gastric intubations showed a darkening of the mucous del cecum and the ascending colon except the group treated with bisacodyl. A degeneration of the cytoplasm and an increase of the apoptosis on the epithelial surface of the colon were observed with all the laxatives. The intestinal changes were more accentuated in the cecum and decreased toward the distal region of the colon. It was concluded that the morphological changes in the large intestine were similar in the treatments with anthranoid and nonanthranoid laxatives, however, the pigments found in the macrophage differed in color and were not always detected macroscopically [26].

In 1968 and 1972 preliminary evidences appeared that led to a certain unease of the potential damaging effect of the stimulating laxatives [27, 28]. These initial investigations proposed that the laxatives containing anthraquinones induced degenerative changes in the nervous tissue of the colon; however, when analyzed by various authors [29–31], these evidences received wide criticism due to the methodology used.

Recent results have undermined the importance of preliminary observations. In rats that were subjected during two years to a treatment with purified senna extract in daily doses of 0, 5, 15, and 25 mg/kg, administered with drinking water, it was discovered that no ultrastructural changes were produced in the myenteric plexus of the colon and jejunum [32].

Although the stimulating laxatives can cause structural damage to the surface of the intestinal epithelial cells, the damage results from an uncertain functional significance; there is no convincing evidence that its chronic use brings as a consequence a structural and/or functional alteration of the enteric nerves or the smooth intestinal muscle [33].

A clinical study was designed to determine the effects of sennosides on the histology of the colon mucosa and in the intestinal preparation for the diagnostic exam [34]. A number of 84 patients received a maximum of 150 mg sennosides A and B, and an intestinal wash was carried out with 3–5 L; the controls only received the intestinal wash solution. The samples from the patients treated with sennosides showed a marked increase in the infiltration of the lamina propria by mononuclear cells. With respect to the tolerance or quality of the intestinal preparation, there were no significant differences. However, because of the microscopic variations that appeared, the use of laxatives containing sennosides was not recommended in patients who were preparing for colonic biopsies [34].

On the other hand, a recent study has concluded that an elevated dose of oral senna is a valid alternative to the conventional use of an intestinal wash solution with polyethylene glycol and electrolytes in the preparation of patients for a colonoscopy. The incidence of adverse effects was similar in both alternatives and patients who used senna commented on less nauseas and vomiting though more abdominal pain [35].

In the necropsy of rats treated for 13 consecutive weeks with a senna preparation, the highest dose of which was 1500 mg/kg/day, a discoloration of the kidneys was observed together with histopathological changes. The latter was observed starting from the 300 mg/kg dose and consisted of a slight-to-moderate tubular basophilia and pigment deposit. This, however, did not correlate to functional changes in the kidney using the determination of set parameters in the laboratory. Likewise, a slight hyperplasia in the large intestine was observed. After an eight-week suspension of the use of the laxative, no histopathological anomalies were found, with the exception of the brown pigmentation of the kidneys [6]. 

Another toxicity study designed a rat treatment with senna fruit extracts 0, 25, 100, and 300 mg/kg/day for 104 weeks. In the necropsy, a dark discoloration was observed on the kidneys of all the groups treated. In the histology, changes in the kidneys of all the animals of the groups treated were observed that included a slight-to-moderate tubular basophilia and pigment deposits. Furthermore, slight-to-moderate hyperplasia was confirmed in the colon and the cecum [21]. These histological changes were reverted once the treatment was suspended [6].

## 5. Melanosis Coli and Apoptosis

A similar condition to the human melanosis coli has been observed in the large intestine of the guinea pig after a daily administration of the anthraquinone danthron. Each treatment provokes a transitory and dose-related wave of apoptosis on the surface of colon epithelial cells. The majority of the apoptotic bodies are phagocytosed by intraepithelial macrophages and transported through the fenestra of the basal epithelial membrane to the lamina propria. Here, the apoptotic bodies transform into typical lipofucsin pigments in the lysosomes of the macrophages. The continuous administration of danthron provokes a progressive accumulation of pigmented macrophages on the intestinal wall while the migration of pigmented macrophages toward the lymphatic nodes region provokes, the sequential loss of the pigmented cells from the superficial and deep lamina propria, once the administration of danthrona ceases, suggesting a similar process in the formation of pigment in man [36].

Melanosis coli is experimentally induced using anthranoid laxatives [37] and has been considered for a long time as a pigmentation that does not provoke damage in the colon and rectum and which is associated with the use of laxatives containing anthraquinones.

In 1997, a retrospective analysis of 2229 patients who used anthraquinone laxatives was carried out in search of a relation between melanosis coli and the use of laxative with the development of colorectal cancer [38]. Although the colorectal adenomas were found with a greater frequency in patients with melanosis than those without, the presence of colorectal cancer was not associated with melanosis coli or the use of laxatives.

The adenomas related with melanosis coli were significantly smaller than those not associated with melanosis coli (*P* < .0001) and were located predominantly in the proximal colon. Differences in the degree of dysplasia or adenomas between patients with or without melanosis coli were not found either [38]. 

It seems there is no association between colorectal cancer and melanosis coli or the use of laxatives. Although the colorectal adenomas are found more frequently in patients with melanosis coli, they do not contain the melanin pigment type. The association of adenomas with melanosis coli can be explained by the easy detection of polyps as white stains in a dark colored colonic mucosa [39].

As has already been indicated, the chronic use of sennoside laxatives on some occasions causes pseudomelanosis coli [40] and this has been associated to an increase in the risk of colorectal cancer [41]. In patients subjected to enemas containing senna extracts, it was observed that the sennosides induce an acute massive loss of intestinal cells, possibly because of apoptosis, and alter the length of the colonic crypts causing the appearance of shorter crypts and an increase of the cellular proliferation and inhibition of the restoring apoptosis of the cellular function. These effects could be the basis of the mechanism that would explain a supposed cancer promoter effect of the chronic use of sennosides [40]. It has been proven that the sennosides administered in an acute manner induce apoptosis of the epithelial cells of the colon, supposedly through the protein p53 “genome guardian,” mediated by p21/WAF, which results in shorter crypts. In conditions where severe melanosis coli was observed, apoptosis seems to be delayed, causing longer crypts without an increase of the proliferating activity or the expression of bcl-2, antiapoptotic gene. It has been suggested that the loss of a possible protective mechanism may increase the risk of carcinogenesis during the chronic use of sennosides [41].

The focus of aberrant crypts is microscopic lesions of the colon mucosa which is suspected to be preneoplastic. The research was carried out to evaluate the cause-effect relation between putative risk factors and colorectal cancer [11, 42, 43]. The anthraquinonic glycosides have been signaled as weak inducers of aberrant crypt foci in the rat colon mucosa [11], and, therefore, weak promoters of carcinogenesis of rat colon. The introduction in the rat diet of high dose of senna after 56 days did not provide evidence of the appearance of aberrant crypt foci and only enhanced the activity of dimethyl hydrazine, known inducer of aberrant crypt foci, when the highest doses of anthraquinonic glucosides were evaluated.

In another study carried out in rats treated daily for 13–28 weeks with 10 mg/kg PO of senna pods extract, a weak laxative effect was observed, which did not provoke an increase of the aberrant crypt foci or tumors in the rat colon; while a diarrheogenic dose (100 mg/kg), capable of inducing chronic diarrhea for three months, increased the appearance of induced tumors by azoximethan known tumor inducer [42]. It is worth mentioning that the usual daily dose in humans is of 0.4 mg/kg. 

In a study carried out in the Universita di Napoli, Italy, and published in 2005, the carcinogenic potential of the anthraquinones used was evaluated using an extract of senna pods. With this objective, healthy rats and rats treated with a tumor-indicating agent of azoximethan were included. The rats were administered 30 and 60 mg/kg of the senna extract for 110 weeks with no development of aberrant crypt foci or tumors in the healthy rats. Surprisingly, the rats that had already been treated with azoximethan reduced the aberrant crypt foci and the tumors, suggesting that a chronic treatment with senna does not predispose to colon cancer and that in contrast, senna could have an antitumoral effect on the colon carcinogenesis [44].

Some years earlier in a review which tried to establish if the stimulating laxatives were harmful for the colon, it was assured that there was no reliable data that connected the chronic use of stimulating laxatives of colorectal cancer and other tumors. In the same article, it was indicated that the risks of stimulating laxatives had been overemphasized and that this had minimized its use by doctors [33].

Another study with patients with sigmoidal cancer and diverticular disease studied the possible association between sigmoidal cancer and constipation, use of anthranoid laxative and melanosis coli using the analysis of aberrant crypt foci as an additional research tool. In the study, 55 surgical patients with sigmoidal cancer were included, 41 surgery patients with diverticular disease, and 96 subjects without intestinal illness. Constipation and use of anthranoid laxatives were similar between patients with sigmoidal cancer (30.9% and 32.7%, resp.) and those with diverticular disease (39% and 26.8%) but greater than in the controls (18.8% and 8.3%). Melanosis coli was found in 38.2% of the patients with sigmoidal cancer and in 39% of those with diverticular disease. The median frequency of aberrant crypt foci was greater in patients with cancer sigmoidal (0.24/cm^2^) than in those with diverticular disease (0.10/cm^2^) and did not vary according to constipation, use of laxatives or melanosis coli in any of the groups. In this study, the hypothesis of a cause-effect relationship of the colorectal cancer with constipation, use of anthranoid laxatives, and melanosis coli was discarded [43].

## 6. Colorectal Cancer

In the Melbourne colorectal cancer study that included 685 cases of cancer colorectal and 723 controls, it was found that chronic constipation self-reported was more common between the cases of cancer than in the controls. Constipation was discarded as a risk factor of intestinal cancer once previously determined dietary risk factors were included, indicating that the diet was associated with the risk of large intestine cancer and not constipation. A greater relationship was established with those that consumed greater amounts of fat. This study concluded that it is unlikely that chronic constipation, diarrhea, and the frequency and consistency of intestinal movement, as well as the use of laxatives, are the etiological factors in the development of colorectal cancer [45].

A retrospective study was carried out in 1993 with 2277 patients following a colonoscopy to determine if the use of laxatives and the existence of melanosis coli diagnosed by endoscopy were risk factors for colorectal neoplasm. Results indicated that there was no increase in the rate of colorectal cancer neither in patients that used laxatives nor in those with melanosis coli. However, there was very significant association between the appearance of colorectal adenomas and use of laxatives (between all patients: 1.72; patients that use laxatives without melanosis coli: 1.47). The relative risk of developing adenoma in patients with melanosis coli was 2.19. Since polyps can be diagnosed easily in the dark mucous of patients with melanosis coli, the authors of this study concluded that the risk of 2.19 seemed to be related to a generalized risk of laxative intake rather than a special group of laxatives that contain anthranoids [46].

A prospective study carried out in the Erlangen University, Germany, investigated the risk of the use of anthranoid laxatives in the development of colorectal adenomas or carcinomas. A total of 202 patients with a recent diagnosis of carcinoma colorectal, 114 patients with adenomatous polyps, and 238 control patients without colorectal neoplasm were included. The use of anthranoid preparations was established in a standard interview, and the existence of melanosis coli visible endoscopically or by microscope was studied by a histopathological exam. There was no statistically significant risk for the use of anthranoids in the development of colorectal adenomas or carcinomas. The presence of macroscopic or microscopic melanosis coli was not a significant risk factor for the development of adenomas or carcinomas. The authors concluded that neither the use of long-term anthranoid laxatives nor melanosis coli were associated with a significant risk of developing colorectal adenoma or carcinoma [47].

In experimental studies carried out in 1993, the effects of the sennosides on cellular proliferation of the epithelium of the ileum and the large intestine were analyzed in rats in vivo. Cell proliferation was examined by the BrdUrd labelling technique after 2, 6, and 12 weeks of continuous treatment. None of the laxatives used, bisacodyl, sennosides, sodium picosulphate, and lactulose, provoked an increase in the cellular proliferation as determined by the techniques used, and the pattern of proliferation remained constant along the crypts. The author concluded the study confirming that the contact laxatives do not have influence on the proliferation of the colon and should not be considered as tumor-promoting substances [48].

Subsequently, another article described that danthrone and sennoside A induced a significant cellular proliferation in the intestine of male F344 rats, after oral administration for 7 days. This effect was dependent on concentration and occurred throughout the intestinal epithelium. Significantly, in the same study, it was indicated that the 1-hidroxianthraquinone induced a slight proliferation of the epithelium in the cecum and colorectum with very little or no toxicity [49]. 

One of the investigators of the Melbourne Colorectal cancer study carried out in Australia in 1988 indicated in 1993 that the analysis of the patients that used laxatives grouped according to the type of laxative used, also allowed the establishment that the prior use of anthraquinone-like laxatives was not associated to colorectal cancer risk either [45]?.

Another study, carried out in rats treated for 2 years with a purified sen extract and which was administered through drinking water, centered on the possible carcinogenicity of this product, placing special emphasis on gastrointestinal alterations. The histopathological exam of the gastrointestinal tract, liver, adrenal kidneys, and other tissues with anomalies did not show differences in the incidence of neoplasic lesions, concluding that there was no relation between long-term administration of a senna extract and the appearance of gastrointestinal tumors or any other type in rat [32].

Another study on carcinogenicity and toxicity designed a treatment of rats with 0, 25, 100, and 300 mg/kg/day of senna fruit extracts for 104 weeks. With the results obtained, it was possible to prove that there was minimal to no hyperplasia in the colon and cecum. Neoplasic changes associated to the treatment with senna were not observed in any of the organs. Based on their results, the authors concluded that senna is not carcinogenic in rats even after a daily administration for two years in doses of up to 300 mg/kg/day [21].

## 7. Mutagenicity, Genotoxicity

The mutagenicity of crude senna preparations and of its glycoside was investigated by Sandnes et al. in 1992 using different strains of *Salmonella typhimurium* [8]. The fruit and leaf senna extracts showed a weak activity in some strains (TA97a, TA100, and TA102). The senna glycosides were inactive in all strains, except for a slight but significant increase in the mutation frequency of TA102. Nonidentified compounds in the crude senna preparations contributed to the low but biologically significant, dose-dependent mutagenicity (greatest in strain TA98). The weak or absent activity of the anthraquinone aglycones in the *Salmonella* strains assayed indicates that the mutagenicity cannot be attributed solely to the anthraquinone content of these plant materials. 

Previously, in 1990 using a variety of structurally related hidroxyanthraquinones, it had been shown that the genotoxicity depends on certain structural requirements [12]. In *Salmonella*, the majority of compounds assayed were mutagenic on strain TA1537, but only the hydroxyanthraquinones with a hydroxymethyl side chain such as lucidin and aloe-emodin were active on other strains. In V79 cells only those with 2 hydroxy groups in the 1, 3 positions such as 1,3-dihydroxyantraquinone, purpurin, and emodin or those with a hydroxymethyl side chain (lucidin and aloe-emodin) were mutagenic.

Other genotoxicity studies have been carried out by various laboratories with senna fructus, senna extract, sennosides, rhein, and aloe-emodin. The first three did not increase the mutation frequency in the following tests: bacterial systems (reverse mutation test in *Salmonella* and/or mutation test in *Escherichia coli*), mammal cell cultures (hypoxanthine guanine phosphorybosiltransferase test, lymphoma test in rat, chromosome aberration test with Chinese hamster ovary cells), medulla osea (micronucleus test, chromosomal aberration test), and melanoblast cells of rats (stain test in rat). Aloe-emodin showed mutagenic effects in vitro in the chromosome aberration test with Chinese hamster ovarian cells and in the *Salmonella* test. In in vivo studies, in the micronuclear test with NMRI rat germ cells, chromosome aberration test with Wistar rat germ cells, and others, there were no indices of mutagenic activity for aloe-emodin. The relevance of the absence of mutagenic potential in in vivo test systems was re-enforced by the fact that aloe-emodin could be found in the sanguine plasma after an oral administration of senna [10].

Senna extracts have been defined as nonmutagenic and as inhibitors of the mutagenicity induced by benzo[a]-pyrene, shamma, aflatoxin B1, and methyl methanosulfonate in the AMES histidine reversion assay using the *Salmonella typhimurium* test strain TA98 [50].

The determination of the genotoxicity profile of senna products, particularly emodin and aloe-emodin in light of other metabolism data in animals and humans or kinetic studies, clinical assays in humans and carcinogenic studies do not support the affirmation that senna-based laxatives mean a genotoxic risk in humans when ingested according to a prescription [51].

Anthraquinones glycosides of *Cassia angustifolia* and *C. fistula* were investigated according to their capacity to induce a clastogenic effect in germ cells of Swiss albino rats. The oral exposure to different doses of anthraquinone and the equivalent quantities in senna leaves and pods did not induce a significant number of chromosomal aberrations or aberrant cells. This reaffirms the concept that anthraquinone sennoside B and rhein are weakly genotoxic [9].

In vivo genotoxic assays of senna extracts, analytically well characterized, have shown that after an oral dose administration of 2,000 mg of senna extract/kg to NMRI, rats of both sexes, equivalent to 119 mg of potential rhein/kg, 5.74 mg of potential aloe-emodin/kg, and 0.28 mg of potential emodin/kg, did not provoke an increase in the levels of micronuclei assay in medulla ossea cells. The clastogenic activity of the senna extract observed in vitro could not be confirmed in the micronucleus assay. The authors of this study concluded that there is no conclusive indication that there is a risk of genotoxicity for patients who consume senna-based laxatives [52].

As a summary, this analysis establishes that (1) there is no convincing evidence that the chronic use of senna brings as a consequence a structural and/or functional alteration of the enteric nerves or of the smooth intestinal muscle, (2) there is no relation between the long-term administration of a senna extract and the appearance of gastrointestinal tumors or of any other type in rat, (3) senna is not carcinogenic in rats even after a daily administration during 2 years in dose up to 300 mg/kg/day, and (4) the evidence currently available does not show that there is a risk of genotoxicity for patients who consume senna-based laxatives.
